# Uncovering the Diversity and Activity of Methylotrophic Methanogens in Freshwater Wetland Soils

**DOI:** 10.1128/mSystems.00320-19

**Published:** 2019-12-03

**Authors:** Adrienne B. Narrowe, Mikayla A. Borton, David W. Hoyt, Garrett J. Smith, Rebecca A. Daly, Jordan C. Angle, Elizabeth K. Eder, Allison R. Wong, Richard A. Wolfe, Alexandra Pappas, Gil Bohrer, Christopher S. Miller, Kelly C. Wrighton

**Affiliations:** a Department of Soil and Crop Sciences, Colorado State University, Fort Collins, Colorado, USA; b Pacific Northwest National Laboratory, Richland, Washington, USA; c Department of Microbiology, The Ohio State University, Columbus, Ohio, USA; d Department of Civil, Environmental & Geodetic Engineering, The Ohio State University, Columbus, Ohio, USA; e Department of Integrative Biology, University of Colorado Denver, Denver, Colorado, USA; Woods Hole Oceanographic Institution

**Keywords:** *Methanomassiliicoccales*, metagenomics, metatranscriptomics, methanol, trimethylamine, wetlands

## Abstract

Wetland soils are one of the largest natural contributors to the emission of methane, a potent greenhouse gas. Currently, microbial contributions to methane emissions from these systems emphasize the roles of acetoclastic and hydrogenotrophic methanogens, while less frequently considering methyl-group substrates (e.g., methanol and methylamines). Here, we integrated laboratory and field experiments to explore the potential for methylotrophic methanogenesis in Old Woman Creek (OWC), a temperate freshwater wetland located in Ohio, USA. We first demonstrated the capacity for methylotrophic methanogenesis in these soils using laboratory soil microcosms amended with trimethylamine. However, subsequent field porewater nuclear magnetic resonance (NMR) analyses to identify methanogenic substrates failed to detect evidence for methylamine compounds in soil porewaters, instead noting the presence of the methylotrophic substrate methanol. Accordingly, our wetland soil-derived metatranscriptomic data indicated that methanol utilization by the *Methanomassiliicoccaceae* was the likely source of methylotrophic methanogenesis. *Methanomassiliicoccaceae* relative contributions to *mcrA* transcripts nearly doubled with depth, accounting for up to 8% of the *mcrA* transcripts in 25-cm-deep soils. Longitudinal 16S rRNA amplicon and *mcrA* gene surveys demonstrated that *Methanomassiliicoccaceae* were stably present over 2 years across lateral and depth gradients in this wetland. Meta-analysis of 16S rRNA sequences similar (>99%) to OWC *Methanomassiliicoccaceae* in public databases revealed a global distribution, with a high representation in terrestrial soils and sediments. Together, our results demonstrate that methylotrophic methanogenesis likely contributes to methane flux from climatically relevant wetland soils.

**IMPORTANCE** Understanding the sources and controls on microbial methane production from wetland soils is critical to global methane emission predictions, particularly in light of changing climatic conditions. Current biogeochemical models of methanogenesis consider only acetoclastic and hydrogenotrophic sources and exclude methylotrophic methanogenesis, potentially underestimating microbial contributions to methane flux. Our multi-omic results demonstrated that methylotrophic methanogens of the family *Methanomassiliicoccaceae* were present and active in a freshwater wetland, with metatranscripts indicating that methanol, not methylamines, was the likely substrate under the conditions measured here. However, laboratory experiments indicated the potential for other methanogens to become enriched in response to trimethylamine, revealing the reservoir of methylotrophic methanogenesis potential residing in these soils. Collectively, our approach used coupled field and laboratory investigations to illuminate metabolisms influencing the terrestrial microbial methane cycle, thereby offering direction for increased realism in predictive process-oriented models of methane flux in wetland soils.

## Abstract (precis)

Understanding the sources and controls on microbial methane production from wetland soils is critical to global methane emission predictions, particularly in light of changing climatic conditions. Current biogeochemical models of methanogenesis consider only acetoclastic and hydrogenotrophic sources and exclude methylotrophic methanogenesis, potentially underestimating microbial contributions to methane flux. Our multi-omic results demonstrated that methylotrophic methanogens of the family *Methanomassiliicoccaceae* were present and active in a freshwater wetland, with metatranscripts indicating that methanol, not methylamines, was the likely substrate under the conditions measured here. However, laboratory experiments indicated the potential for other methanogens to become enriched in response to trimethylamine, revealing the reservoir of methylotrophic methanogenesis potential residing in these soils. Collectively, our approach used coupled field and laboratory investigations to illuminate metabolisms influencing the terrestrial microbial methane cycle, thereby offering direction for increased realism in predictive process-oriented models of methane flux in wetland soils.

## INTRODUCTION

Wetlands are the largest natural source of atmospheric methane, one of the most potent greenhouse gases contributing to global climate change (1). Identifying the source of this methane through interrogation of the below-ground microbial processes in soils is critical to accurately forecasting methane emissions today and in the future. Methane-producing *Archaea*, methanogens, use a narrow range of substrates for methane production, including acetate, hydrogen/CO_2_, or methylated compounds (e.g., methylamines and methanol) (2). Of these, methylotrophic methanogenesis is commonly recognized as critical to the methane cycle in sulfate-rich and/or saline systems (3–5) and, more recently, in a freshwater peatland system (6). However, the substrate profiles, identity, distribution, and activity of methylotrophic methanogens are less frequently considered in freshwater, nonpeat wetland soils (7–10). This knowledge gap contributes to the fact that contemporary process-based biogeochemical models account only for microbial methane production from acetate and hydrogen in soil systems (11, 12).

Methylotrophic methanogenesis results from the demethylation of methyl-group (C_1_)-containing compounds including methanol as well as trimethylamine, dimethylamine, and monomethylamine (TMA, DMA, and MMA, respectively) (13). In wetland soils, methanol can be produced from decomposition of plant-derived lignin and pectin (14). Methylamine compounds may also originate from plants in wetlands, as they can be derived from quaternary amines (e.g., choline, carnitine, and glycine betaine) which are common plant exudates and osmoprotectants (15). Recent metagenomic investigations have demonstrated that methylotrophic methanogenesis may be more prevalent and phylogenetically diverse than previously recognized across a range of habitats, renewing interest in the environmental distribution and contribution of this metabolism to the global carbon cycle (16, 17).

Here, we characterized the potential for methylotrophic methanogenesis in freshwater wetland soils from Old Woman Creek (OWC), a National Estuarine Research Reserve, located in Ohio, USA. Previous work showed that OWC had annual mean methane emissions reaching 82 g CH_4_-C m^−2^ (18, 19). Moreover, our prior research, from the same year that samples in this study were collected, demonstrated that the dominant methanogen in surface (0- to 5-cm) soils was a member of the acetoclastic *Methanothrix* (20). Contrary to long-held assumptions about the environmental constraints on methanogenesis, this *Methanothrix* archaeon accounted for nearly 90% of the *mcrA* transcripts in these bulk oxygenated soils, where up to 80% of methane was inferred to originate (20). Notably, 16S rRNA surveys revealed that other methanogenic taxa, including members of the methylotrophic *Methanomassiliicoccaceae*, were also present in these wetland soils (20, 21). This finding, combined with results from peatland soils (6, 22, 23), raised the possibility that methylotrophic methanogenesis could contribute to methane flux in this and other freshwater wetlands, particularly in light of changing environmental conditions (e.g., plant cover). In this study, we analyzed soil porewater metabolite data and metagenomic and metatranscriptomic data from laboratory microcosm and field experiments to define the substrate profiles as well as the phylogeny and metabolisms of methylotrophic methanogens in OWC soils. This combined laboratory and field data set is a necessary step to uncovering methylotrophic diversity and metabolisms that can contribute to methane flux across terrestrial biomes.

## RESULTS AND DISCUSSION

### Experimental design.

**FIG 1:**
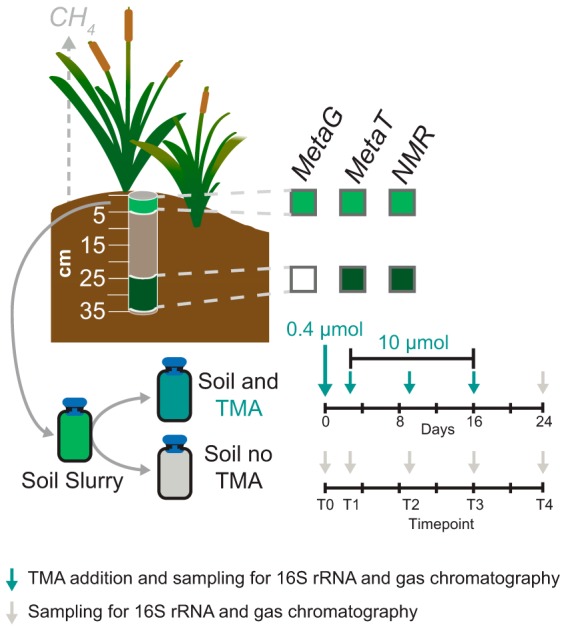
Experimental design for culture-dependent and independent description of methylotrophic methanogenesis in wetland soils. A soil core from a plant-covered wetland site was subsectioned by depth. The 0- to 5-cm and 24- to 35-cm sections were used for ^1^H NMR identification of porewater metabolites and for shotgun metatranscriptomic sequencing. Triplicate soil microcosms were either amended with TMA or unamended (endogenous controls). Methane production and microbial community composition were characterized approximately weekly over 24 days as indicated by arrows along the timeline. TMA was added at time points 0 to 3 to reach the in-tube concentrations shown.

To investigate the prevalence and activity of methylotrophic methanogenesis in wetland soils, we performed both culture-dependent and field-scale multi-omics assays on OWC wetland soils. All soils investigated in this publication were collected from the same 1-m^2^ plot beneath a patch of emergent vegetation, Typha latifolia (Fig. 1). To first assay the potential for methylotrophic methanogenesis in these soils, bulk surface (0- to 5-cm) soils where we previously observed the highest methane production (20) were used as an inoculum for laboratory assays. Triplicate anoxic soil microcosms were amended with and without trimethylamine (TMA) and incubated at field-relevant temperature for 24 days, with samples for 16S rRNA gene analysis and methane production taken prior to each amendment, while H-nuclear magnetic resonance (NMR) metabolites were analyzed on the initial and final time points (Fig. 1). TMA, rather than methanol, was chosen as the substrate for the microcosms as it was inferred to be a more noncompetitive substrate for the surrounding soil microbial community (24, 25). To better understand which methylotrophic methanogens were present and active under field-relevant conditions, we analyzed soil multi-omic data from the same field-collected soils used as the inoculum for the laboratory microcosms. Specifically, metatranscriptomic and H-NMR-detected metabolite data were recovered from surface (0- to 5-cm) and deep (24- to 35-cm) soils and porewaters, respectively, collected from the *Typha* plot and mapped to paired metagenomic data recovered from the wetland (Fig. 1).

### Trimethylamine amendment stimulates methane production in freshwater wetland soils.

**FIG 2:**
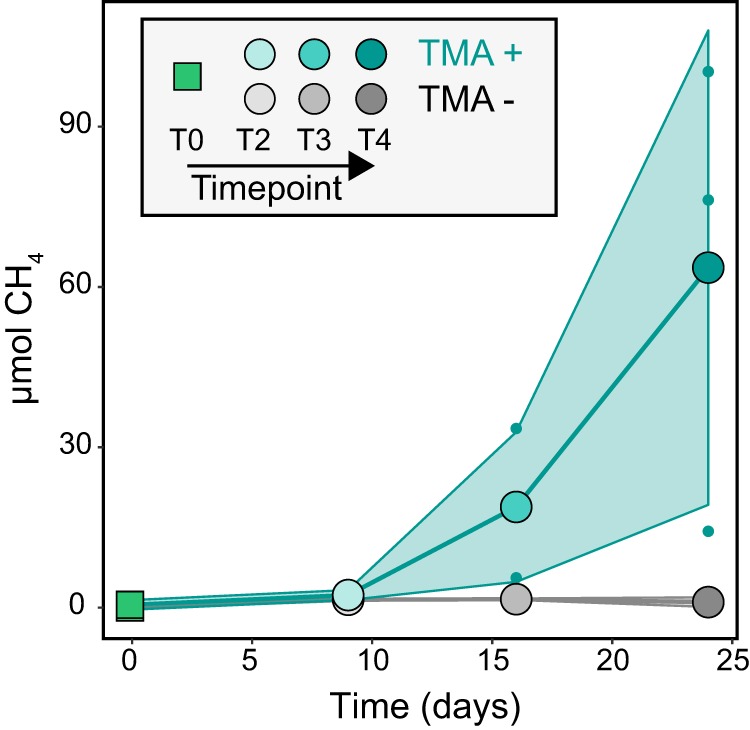
TMA amendment of wetland soils demonstrates the potential for methylotrophic methanogenesis. Methane production rates for TMA-amended samples (teal) exceeded that of unamended controls (gray) and increased over the course of the 4-week incubation. Filled circles show mean methane emission for triplicate samples, and small dots show individual values for the 3 samples. The shaded area encloses the area within 1 SD of mean emission rate.

Microcosm experiments showed that TMA-amended soils produced on average 41-fold greater total methane than TMA-unamended soils (Fig. 2; see also Table S1 in the supplemental material). By the end of the incubation experiment, the TMA-amended soils had an average of 63.62 μmol methane produced in the headspace, while unamended soils produced on average 1.03 μmol methane from endogenous substrates (Fig. 2 and Table S1). These results indicated that the potential for rapid and substantial methane production from methylotrophic substrates exists within these freshwater wetland soils.

10.1128/mSystems.00320-19.3 TABLE S1 Microcosm methane measurements. Download Table S1, PDF file, 0.03 MB. Copyright © 2019 Narrowe et al. 2019 Narrowe et al. This content is distributed under the terms of the Creative Commons Attribution 4.0 International license.

Metabolite analysis of the microcosms at the initial and final time points of the incubation experiment indicated that added TMA was fully consumed, as no TMA was detected after 24 days. Moreover, most subsequent demethylation products of TMA were also absent, with DMA below detection at the final time point and MMA below detection in 2 of the 3 amended microcosms (1 microcosm had a final concentration of 49.5 μM) (Table S2). However, similarly to previously reported experiments with anaerobic soil microcosms amended with methylamines (5), acetate significantly increased from a mean of 9 μM in the initial time point to 27.9 μM in the unamended microcosm and 241.1 μM in the TMA-amended microcosms at the final time point (Table S2). This increase in acetate production in the TMA-amended microcosms could be from demethylation by nonmethylotrophic methanogenic processes, as this demethylation has been observed with both aerobic and anaerobic bacteria (26, 27). Thus, while our findings showed that TMA stimulated methane production from these OWC wetland soils, it is not clear if the methane was produced directly from TMA via methylotrophic methanogenesis or indirectly via acetoclastic methanogenesis. The capacity for TMA to support methane production has not been consistently reported from other wetland soils or lake sediment microcosms (6, 23, 28–31). Explanations for differences in methane production in anoxic soil microcosms highlight the need to understand the diversity and substrate specificity of methylotrophic organisms in soil systems (27, 32, 33).

10.1128/mSystems.00320-19.4 TABLE S2 H-NMR and ion chromatography results for field soil samples and microcosms. Download Table S2, PDF file, 0.07 MB. Copyright © 2019 Narrowe et al. 2019 Narrowe et al. This content is distributed under the terms of the Creative Commons Attribution 4.0 International license.

To investigate the microbial community response to TMA amendment, we monitored microbial membership and community structure using 16S rRNA gene amplicon sequencing of our amended and unamended microcosms approximately weekly for 24 days (Data Set S1). TMA-amended soils had significantly different microbial communities than unamended soils (Fig. S1). The sole discriminating archaeal taxon for TMA amendment was the genus *Methanosarcina* (Data Set S2), a member of the *Methanosarcinales* known for broad substrate use, including methylamines, methanol, hydrogen, and acetate (34). This taxon increased over 100-fold from the inoculum and was significantly enriched with time and relative to the control final time point (linear discriminant analysis [LDA] 4.5, *P* = 0.04). While 16S rRNA gene sequences associated with methanogens related to methylotrophic *Methanomassiliicoccaceae*, *Methanolobus*, and *Methanomethylovorans* and acetoclastic *Methanothrix* were detected in TMA-amended soils over time, none were discriminant taxa between the two treatments. Notably, members of the *Methanosarcinales* were rare members of the wetland soil community (in many cases at the detection limit) and detected in only 54% of 150 soil samples collected over a 2-year period (20, 21, 35), where the dominant methanogen (*Methanothrix*) was detected in 76% of the 150 samples (20, 21, 35) and accounted for up to 47% of the archaeal community (21). In contrast, other methylotrophic methanogens detected in the enrichments (namely, *Methanomassiliicoccaceae*) were present in higher abundances in the field (reaching 15% of the archaeal community [21]) and detected in 77% of the 150 field samples (20, 21, 35).

10.1128/mSystems.00320-19.1 FIG S1 Nonmetric dimensional scaling (NMDS) ordination of intersample Bray-Curtis dissimilarity. Control (no TMA amendment) samples clustered by replicate regardless of time point (MRPP A = 0.07, *P* = 0.0002), while all TMA-amended samples showed similar changes in microbial community composition and were statistically different from control samples by the final two time points (MRPP A = 0.07, *P* = 0.0005). Arrows connect same-replicate samples with the arrowhead indicating increasing incubation time. Initial inoculum samples are shown in green. *T*_0_, initial inoculum. *T*_2_, *T*_3_, and *T*_4_ indicate subsequent time points. *T*_1_ samples did not yield sufficient DNA for 16S rRNA sequencing and are not shown. Download FIG S1, PDF file, 0.2 MB. Copyright © 2019 Narrowe et al. 2019 Narrowe et al. This content is distributed under the terms of the Creative Commons Attribution 4.0 International license.

10.1128/mSystems.00320-19.7 DATA SET S1 Microcosm 16S rRNA amplicon ASV table. Download Data Set S1, TXT file, 0.9 MB. Copyright © 2019 Narrowe et al. 2019 Narrowe et al. This content is distributed under the terms of the Creative Commons Attribution 4.0 International license.

10.1128/mSystems.00320-19.8 DATA SET S2 LEfSe linear discriminant analysis results. Download Data Set S2, TXT file, 0.3 MB. Copyright © 2019 Narrowe et al. 2019 Narrowe et al. This content is distributed under the terms of the Creative Commons Attribution 4.0 International license.

Given the challenges of recreating the habitat heterogeneity in soils effectively in the laboratory, the differences between microbial members enriched in field and laboratory soil microcosms are not necessarily unexpected (36, 37). Factors contributing to these differences could include discrepancies between *in vivo* and *in vitro* soil nutrient or substrate concentrations (38, 39), the freezer storage of these soils prior to lab inoculation (see Materials and Methods), or the lack of coamendment with hydrogen, as all cultivated *Methanomassiliicoccaceae* to date require this electron donor in addition to the methylated substrate (40–43). Alternatively, the laboratory enrichment of taxa that are rare under currently measured field conditions highlights the vast, undersampled, yet conditionally active methanogenic capacity residing in these diverse wetland soils. As others have suggested (44), this untapped methanogenic potential may be important for ecosystem functional stability under changing environmental conditions.

### Substrate profiles and metatranscripts provide evidence supporting methylotrophic methanogen activity in wetland soils.

We profiled methanogenic substrate concentrations from porewaters collected from surface and deep soils in the *Typha* site, the same soil core used as an inoculum source for our laboratory microcosms (Fig. 1) (20). Substrates that support methylotrophic and acetoclastic methanogenesis were detectable and distributed along a depth gradient in replicate soil cores from this site. Consistent with what we previously reported (20), acetate concentrations in these *Typha* wetland soils were 2-fold higher in the surface than the deep soils (Table S2). In contrast to acetate, which was greater in the oxygenated surface soils (0 to 5 cm), methylotrophic substrates, namely, methanol, were higher in concentration in the anoxic, deep soils (24 to 35 cm) (Table S2) (20).

Of the methylotrophic substrates queried (TMA, DMA, MMA, and methanol), methanol was measured at 23-times-greater concentrations in deep soils relative to surface soils, and with concentrations approaching or exceeding 1 mM (mean 989 ± 684 μM) (Table S2). Our methanol concentrations were similar to reports from temperate peat soils (490 μM) (23) and high-altitude wetland peat soils (460 to 2,800 μM) (30), which showed that methane production from methanol could be sustained across saturated peat soils. Notably, here methylamines and formate were not measured above detection limits of 1 μM. Yet, even low methylamine concentrations have been shown to support methane production, as TMA concentrations in methane-emitting lake sediments of 0.2 to 2.2 μM were near or below the 1 μM detection threshold employed here (28). Consequently, based on substrate profiles alone, the biological usage of these potential substrates in supporting methylotrophic methanogenesis in these soils could not be inferred.

**FIG 3:**
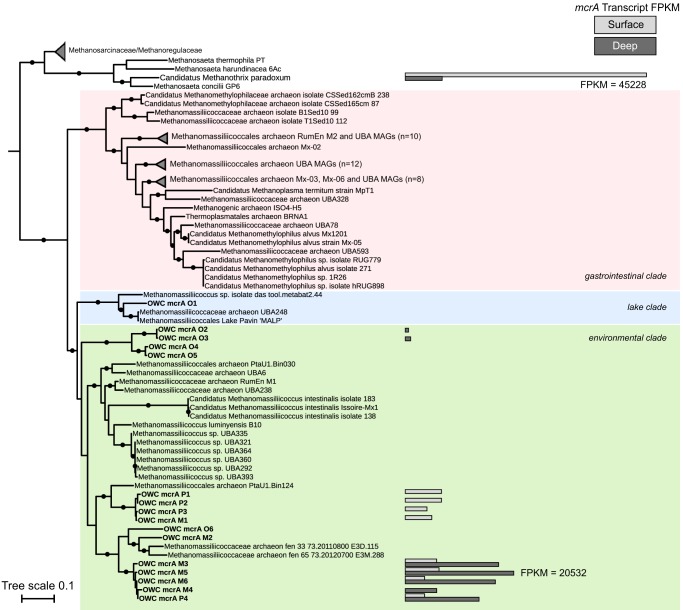
Transcription of *mcrA* genes in wetland soils shows a diversity of active methanogens. Maximum likelihood phylogeny of *Methanomassiliicoccaceae mcrA* genes from wetland soils with sequences from genome-sequenced members included for reference. Transcription of *mcrA* genes as measured by mapping of metatranscriptomic sequencing reads to gene sequences is shown in bars. Light gray represents mean transcription in surface soils (*n* = 3), and dark gray represents mean transcription in deep soils (*n* = 3). Normalized transcription is measured as FPKM (fragments per kilobase of transcript per million reads mapped). “*Candidatus* Methanothrix paradoxum” from this wetland is shown as a reference (20). UFBoot branch support values of ≥95 are shown by filled circles. Clade classification is based on the placement of genomes within the environmental, gastrointestinal, and lake clades as defined by Söllinger et al. (48) and Speth and Orphan (49).

Using the methyl coenzyme M reductase alpha subunit (*mcrA*) gene, a hallmark for inferring diversity and activity of methanogenesis and anaerobic methane oxidation (45, 46), we investigated methylotrophic methanogen presence and activity in surface and deep wetland soils (Fig. 3). From metagenomic data collected over 2 sampling years, three wetland land cover sites, and multiple soil depths (*n* = 11 total metagenomes) (Table S3), we reconstructed 6 full-length and 10 partial *mcrA* genes belonging to the family *Methanomassiliicoccaceae* (Data Set S3). However, consistent with our 16S rRNA gene field surveys, *Methanosarcina* was often at or below the detection limit in these wetland soils, with only a single *mcrA* gene in our broader wetland *mcrA* gene database assigned to *Methanosarcina*.

10.1128/mSystems.00320-19.5 TABLE S3 Metagenome and metatranscriptome information. Download Table S3, PDF file, 0.04 MB. Copyright © 2019 Narrowe et al. 2019 Narrowe et al. This content is distributed under the terms of the Creative Commons Attribution 4.0 International license.

10.1128/mSystems.00320-19.9 DATA SET S3 Fasta file of *Methanomassiliicoccaceae mcrA* sequences and transcribed *mtaB* sequences. Download Data Set S3, TXT file, 0.03 MB. Copyright © 2019 Narrowe et al. 2019 Narrowe et al. This content is distributed under the terms of the Creative Commons Attribution 4.0 International license.

By mapping metatranscriptome reads from the surface and deep layer of cores collected at the *Typha*-covered site to this *mcrA* gene database, we showed that methylotrophic methanogens belonging to the *Methanomassiliicoccaceae* were active along the soil depth profile (Fig. 3 and Data Set S4). We previously reported the high level of transcription of acetoclastic “*Candidatus* Methanothrix paradoxum” *mcrA* genes, which dominated surface soil transcript levels (20) (Fig. 3). In contrast, in deep soils the relative percentage of acetoclastic methanogen *mcrA* transcripts decreased, concomitant with increases in methylotrophic *Methanomassiliicoccaceae mcrA* transcripts, which accounted for up to 8% of the *mcrA* transcripts. *Methanomassiliicoccaceae mcrA* transcription in both surface and deep soils suggests that methylotrophic methanogenesis may contribute to the methane cycle in this system (Fig. 3).

10.1128/mSystems.00320-19.10 DATA SET S4 FPKM/TPM data for reported genes. Download Data Set S4, XLS file, 0.03 MB. Copyright © 2019 Narrowe et al. 2019 Narrowe et al. This content is distributed under the terms of the Creative Commons Attribution 4.0 International license.

### OWC strains resolve depth-differentiated lineages within the *Methanomassiliicoccaceae* environmental clade.

As initially proposed by Paul et al. (47), Söllinger et al. used 16S rRNA gene and *mcrA* gene phylogenetic analyses to conclude that the *Methanomassiliicoccaceae* can be assigned to two clades that included a gastrointestinal clade and an environmental clade (48–50). These findings were more recently supported by Speth and Orphan (49) using the *mcrA* gene, also confirming a third, lake sediment cluster as suggested earlier by Borrel et al. (50). Here, we placed our OWC sequences in the context of these three previously defined *Methanomassiliicoccaceae mcrA* lineages, referring to them as gastrointestinal, lake, and environmental clades (Fig. 3). One OWC *Methanomassiliicoccaceae mcrA* sequence was placed within the lake clade while the remaining 15 OWC *Methanomassiliicoccaceae mcrA* sequences clustered with members in the environmental clade. The majority of the OWC sequences were distinct from the 20 previously described *Methanomassiliicoccaceae* genomes and metagenome-assembled genomes (MAGs) in the lake and environmental clades. The closest genomic representatives to the OWC *mcrA* gene sequences are from a MAG (PtaU1_Bin124, 90% nucleotide identity to OWC1 clade) reconstructed from a methanogenic sludge blanket reactor amended with aromatic compounds (51) and a MAG reconstructed from Stordalen mire, a thawing permafrost fen (Methanomassiliicoccaceae_archaeon_isolate_fen_33_73.20110800_E3D.115, 89% nucleotide identity to OWC2 clade [B. J. Woodcroft, C. M. Singleton, and J. A. Boyd, unpublished data]) (Fig. 3). Our findings revealed the considerable diversity within the OWC *Methanomassiliicoccaceae*, as the *mcrA* genes we sampled form three distinct, well-supported clades within this environmental cluster (Fig. 3). This suggests that high diversity is maintained for a single ecosystem function (e.g., methanol-driven methanogenesis [52]).

We further explore the notion that strain variation revealed in the *mcrA* gene could reflect niche partitioning at the strain or clade level. In support of this, we note that members of the three OWC clades exhibited distinct depth-resolved *mcrA* transcriptional patterns. For instance, members in two clades (OWC1 and OWC2) exhibited transcription in either the surface or the deep metatranscriptome only, while 4 of 5 sequences in the OWC3 clade were transcribed at both soil depths (Fig. 3). Those *Methanomassiliicoccaceae mcrA* genes that were transcribed in both depths were consistently more highly transcribed in the deep soils. We consider that the increased levels of *mcrA* transcription in the deeper soils for the *Methanomassiliicoccaceae* could be due to habitat compatibility, substrate availability, or lack of resource competition from other taxa which thrive in the surface soils. Moreover, our soil metatranscriptomics show that closely related strains can have variable activity along depth and redox gradients in a mineral soil freshwater wetland.

### Methanol is inferred to be the active methylotrophic substrate.

To better investigate which substrates support methylotrophic methanogenesis in this wetland, we queried the metagenomes and metatranscriptomes for functional genes specifically associated with the use of methylotrophic methanogenesis substrates. We detected only limited capacity for methanogenesis via methylamines in our field-derived multi-omic data. For example, in the plant soil metagenome, pyrrolysine-containing monomethylamine or trimethylamine methyltransferases (*mtmB* and *mttB*), essential for methylotrophic methanogenesis from methylamine and trimethylamine, respectively, were not detected. The metagenome did contain 3 putative dimethylamine methyltransferase (*mtbB*) genes; however, we detected no transcription of these methylamine-specific genes in either the surface or deep metatranscript libraries (*n* = 6), indicating that methylamines in the wetland soils were likely not directly supporting methanogenesis under these sample conditions. These metatranscriptome-based findings are in agreement with the lack of detectable methylamines that we observed in our porewater metabolite analysis.

**FIG 4:**
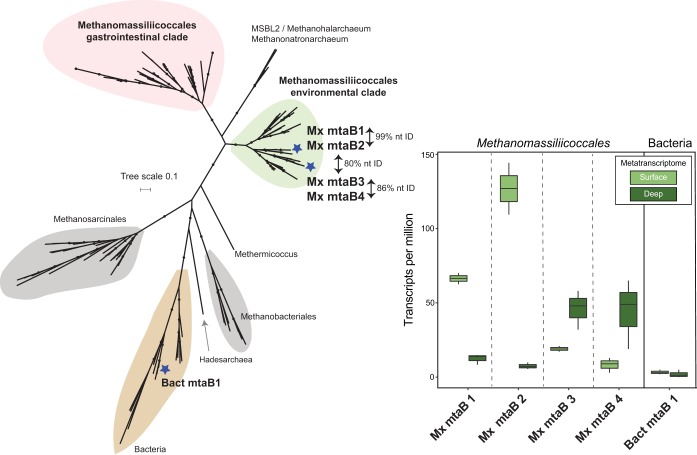
*Methanomassiliicoccaceae mtaB* genes in wetland soils show depth-differentiated transcription profiles. (Left) Maximum likelihood *mtaB* gene phylogeny places transcribed *mtaB* from OWC metagenomes within the *Methanomassiliicoccaceae* environmental clade in agreement with Fig. 3. An additional *mtaB* gene sequence was associated with a clade of bacterial *mtaB* genes. (Right) Box plots showing the transcription (transcripts per million) of methanol methyltransferase (*mtaB*) genes in both surface (0- to 5-cm) and deep (24- to 35-cm) wetland soil metatranscriptomes.

In contrast to methylamine-utilizing genes, genes for the use of methanol (*mtaB*, methanol-5-hydroxybenzimidazolylcobamide comethyltransferase) were both present and transcribed in the shallow and deeper soils. Phylogenetic analysis placed four of five OWC metagenomic *mtaB* genes within a clade comprised of *Methanomassiliicoccaceae mtaB* gene sequences (Fig. 4). In agreement with the *mcrA* gene phylogeny (Fig. 3), here we report that the *Methanomassiliicoccaceae mtaB* gene sequences formed two clades corresponding to the environmental and gastrointestinal clades. The four transcribed *Methanomassiliicoccaceae mtaB* genes from the OWC metagenomes derived from different soil depths and land coverage types (Data Set S3). Two of the *Methanomassiliicoccaceae* genes (Mx *mtaB* 1 and Mx *mtaB* 2) were highly similar (>99% nucleotide identity) and may derive from closely related strains; however, there was no more than 80% nucleotide identity among these and the other *Methanomassiliicoccaceae mtaB* genes from this wetland (Fig. 4). All four *Methanomassiliicoccaceae mtaB* genes recruited transcripts from both surface and deep soils (Fig. 4). This recruitment of the *mtaB* transcripts across soil depths also agreed with the patterns observed for the *mcrA* genes, suggesting that multiple depth-defined *Methanomassiliicoccaceae* organisms inhabit and are active in wetland soils (Fig. 4 and Data Set S4).

In addition to the methanogens, we also examined taxa potentially competing for methanol utilization. We recovered bacterial *mtaB* and bacterial methanol-corrinoid protein (*mtaC*) genes, suggesting the potential for anaerobic methanol utilization by bacteria (27, 53, 54). However, only 2 of these bacterial *mtaBC* genes recruited transcripts and did so at comparatively lower levels than the *Methanomassiliicoccaceae* methanol-utilizing genes, suggesting that significant competition from anaerobic bacterial methanol oxidizers was unlikely during the time of sampling (Fig. 4 and Data Set S4). In comparison, since oxygen and other electron acceptors like ferric iron and nitrate were detectable in our surface soils (20), we also investigated the presence and activity of methyltroph and nonmethylotroph methanol dehydrogenase genes that could couple methanol oxidation to the other available electron acceptors in these soils (21, 35). We detected multiple *xoxF* homologs in the metagenomes. Of those 9 genes that were transcribed, 7 derived from one of the most dominant taxa in our samples, and the most active methanotroph, a member of the genus *Methylobacter* (35) (Data Set S4). In addition to these *xoxF* homologs, we also identified 4 bacterial pyrroloquinoline quinone (PQQ)-dependent alcohol dehydrogenases which were transcribed in the surface soils and suggest competition for methanol (25). However, future investigations using isotopically labeled methanol will be necessary to determine the metabolic fate of methanol in the oxic soil layer.

Our meta-omics data suggested that the *Methanomassiliicoccaceae* utilized methanol rather than methylamines. This finding agrees with a recent analysis of a *Methanomassiliicoccales* MAG from a freshwater lake (Lake Pavin MALP [Fig. 3]) that similarly failed to find evidence of methylamine methyltransferases in the genome, recovering instead only *mtaBC* genes for methanogenesis from methanol (49). A similar absence of methylamine methyltransferases was reported for an additional *Methanomassiliicoccaceae* MAG from rumen fluid enrichments (RumEn M2 [Fig. 3]), with a second enrichment MAG containing only a single methylamine methyltransferase (RumEn M1 [Fig. 3]) (48).

Unlike other methylotrophic methanogens, e.g., *Methanosarcina*, the cultivated *Methanomassiliicoccaceae*, which have all been isolated from gut ecosystems to date, require H_2_ as an electron donor in addition to the methylated substrate (40–43). From our data set, we did recover the metabolic potential for hydrogen metabolism from at least three different *Methanomassiliicoccaceae* strains. These hydrogenase genes, *hdrA2B2C2* (EC 1.8.7.3), and *mvhD* (EC 1.12.99) had best hits to sludge reactor metagenome *Methanomassiliicoccaceae* (bit scores 81 to 593). Notably, while we did detect expression of methanol-utilizing genes, we failed to recruit transcripts to these hydrogenase genes, leaving open the possibility that hydrogen requirements for these field *Methanomassiliicoccaceae* may differ from those of the gut-derived strains.

To begin to evaluate if methylotrophic substrate use followed phylogenetic or environmental patterns, we searched 103 publicly available *Methanomassiliicoccales* genomes in GenBank for homologs of methylamine and methanol methyltransferases. Of the 70 available genomes/MAGs that contained an *mcrA* gene, we found that 70% of the genomes/MAGs in the gastrointestinal clade contained homologs of genes for methylamine utilization compared to only 45% of genomes/MAGs in the environmental and lake clades (Fig. S2). In contrast, homologs of methanol methyltransferases were detected in only 76% of the gastrointestinal clade, while they were detected in 95% of the environmental and lake clade genomes/MAGs. These findings suggest that the methylotrophic substrate utilization patterns may vary across this methanogen order, though some of these apparent absences may be the result of incomplete genome assemblies. Clearly, more cultured representatives, especially from environmental clades, will be necessary to elucidate the true methylotrophic substrate range of this methanogenic order.

10.1128/mSystems.00320-19.2 FIG S2 Presence of methylamine and methanol methyltransferases in *Methanomassiliicoccales* genomes and MAGs. Maximum likelihood phylogeny of *mcrA* genes as shown in Fig. 3. Here, the label colors indicate the presence of methylamine methyltransferase homologs (*mttB*, *mtbB*, and *mtmB*) and blue branch colors indicate the presence of methanol methyltransferase (*mtaB*) homologs in publicly available genomes. Download FIG S2, PDF file, 2.0 MB. Copyright © 2019 Narrowe et al. 2019 Narrowe et al. This content is distributed under the terms of the Creative Commons Attribution 4.0 International license.

### Methanogenesis from methanol may represent a broadly distributed component of the carbon cycle in wetland soils.

**FIG 5:**
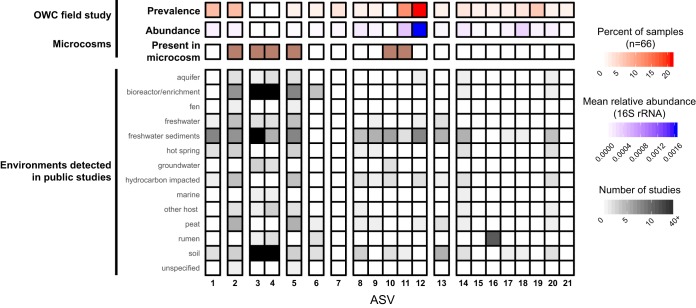
Broad environmental distribution of *Methanomassiliicoccaceae* 16S rRNA sequences includes high prevalence in freshwater ecosystems. The 21 ASVs from the field and from microcosms from this study are grouped by sequence homology (>98% nucleotide identity). For the field-derived ASVs, red shows occupancy as a percentage of the 66 samples in which each ASV was detected, and blue shows the ASV mean relative abundance within those 66 samples. Brown indicates those ASVs detected in 16S rRNA sequencing of microcosm slurries. Grayscale indicates the number of studies for each environmental type for which each OWC ASV had at least one qualified hit (see Materials and Methods). The 5 blocks shown in black indicate sequences found in >40 studies (*n* = 42 to 83).

We performed a meta-analysis of the 21 *Methanomassiliicoccaceae* amplicon sequence variants (ASVs) identified in this wetland over a 2-year, multiseason sampling campaign and our laboratory microcosms (*n* = 87 samples) to determine their local and global distribution patterns. In the Old Woman Creek wetland soils, the *Methanomassiliicoccaceae* comprised a core component of the methanogen community, reaching up to 15% of the archaea as estimated by 16S rRNA gene relative abundance (21). Issues such as PCR primer bias and differing 16S rRNA gene copy numbers prevent direct inference of total community abundances; however, a similar proportional abundance was also observed in metagenomic sequencing in a prior analysis of read-mapping to single-copy genes (see supplementary figure 9 in the work of Angle et al.) (20). Analysis performed here shows that *Methanomassiliicoccaceae* were distributed laterally throughout the wetland, beneath multiple different land coverage types, including vegetation (*Typha*), seasonal mud flats, and persistently submerged sites. They were also present along the soil depth profile and stably maintained during more than 2 years of sampling in this wetland (21, 35) (Fig. 5). Interestingly, the most prevalent and abundant ASV in our field samples (ASV 12) did not persist in the microcosms and had a narrow environmental range, primarily occurring in studies from freshwater sediments. In contrast, *Methanomassiliicoccaceae* ASVs 3 and 4, which were among the most abundant in the microcosm, were not detected in our field samples during the 2014–2015 sampling but were more broadly distributed environmentally, appearing in natural as well as engineered samples (e.g., bioreactor and enrichment cultures) (Fig. 5). Here, too, our findings demonstrate the importance of integrating -omics and laboratory methods, especially for sampling the taxonomic breadth of methanogens, which are often low-abundance members in highly diverse soil communities (36, 55).

### Conclusions.

Relative to acetoclastic and hydrogenotrophic methanogenesis, methylotrophic methanogenesis is comparatively less studied in freshwater terrestrial ecosystems (7–10). This lack of information may have ramifications, especially in climatically relevant, methane-emitting ecosystems like saturated natural or agricultural soils, as well as sediments from inland lake and river waters (56). Here, we used cultivation-based and cultivation-independent approaches to define the microbial members performing methylotrophic methanogenesis in freshwater wetland soils. Laboratory soil microcosms amended with TMA enriched *Methanosarcina* sp. methanogens despite their apparent inactivity in the field under the currently measured conditions. This suggests additional latent potential for methanogenesis stored in wetland soils and highlights the importance of cross-validating laboratory and field experiments. In contrast, field metatranscriptome data suggested that methanol, rather than methylamines, may be supporting the methylotrophic *Methanomassiliicoccus* methanogens active in these wetland soils.

Quantifying the contribution of methanol to the methane production and flux from freshwater soils will ultimately require the use of stable-isotope analyses (57). With this method, methanol was estimated to be the basis of up to 5% of the methane produced in a freshwater lake measured at a single time point (28). While this estimate is substantially lower than contributions from acetoclastic or hydrogenotrophic pathways, net methane emissions from wetlands systems are large. If similar contributions are demonstrated in soils, then the overall contribution of methanol-derived methanogenesis from wetland soils could contribute significantly to atmospheric methane concentrations (23, 28–30, 58, 59). In summary, the broad environmental distribution of *Methanomassiliicoccaceae*, combined with measured availability of substrates and activity, suggest that methanol-based methanogenesis by this order should be considered a potential component of the global methane cycle in freshwater wetland soils.

## MATERIALS AND METHODS

### Experimental design and sample collection.

In this study, a single soil sample collected from a plant-covered mudflat (August 2015) was used to build microcosms to assess the potential for methylotrophic methanogenesis in wetland soils. Soil cores were collected from the Old Woman Creek National Estuarine Research Reserve (OWC) (41°22′N 82°30′W) as described previously during the month of August 2015 from a plant-covered mudflat (20). Soil samples were stored at −20°C until use in the microcosm experiment. In order to match the microcosm experiments with the field data, it was necessary to use frozen samples. We also note that water and air temperatures at OWC routinely fall below 0°C and approach or fall below −20°C, respectively, throughout the winter months, but that freezing and thawing of the soils in the laboratory may have impacted the viability of certain methanogen types. The microcosm experiment consisted of 3 treatments: treatment 1, trimethylamine and soil; 2, no trimethylamine and soil (no-substrate control); and 3, trimethylamine and no soil (medium control). As expected, the abiotic treatment (no soil addition) showed no methane production and had no DNA recovery over the 24-day experiment. Trimethylamine, as opposed to methanol, was selected as a model methylotrophic substrate, as it was assumed to be less susceptible to cross-feeding nonmethanogen members in these oxygenic, surface soils (24, 25, 35). Treatments 1 and 2 were generated in triplicate, while treatment 3 was done singly. For treatments 1 and 2, each tube consisted of 10% anoxic soil slurry and 90% sterile basal bicarbonate-buffered medium dispensed in Balch tubes sealed (10 ml) with butyl rubber stoppers and aluminum crimps under an atmosphere of N_2_-CO_2_ (80:20, vol/vol). Before mixing with soil slurry, the medium (per liter) included 0.25 g ammonium chloride, 0.60 g sodium phosphate, 0.10 potassium chloride, 2.5 g sodium bicarbonate, 10 ml dl-vitamin mixture (see Table S4 in the supplemental material), and 10 ml dl-mineral mixture and was brought to a pH of 7.0 using 1 mM NaOH. Tubes were incubated at 25°C, which is consistent with the measured temperature of these soils in the field (25.0°C for surface soils 0 to 5 cm [Table S2]).

10.1128/mSystems.00320-19.6 TABLE S4 Microcosm medium composition. Download Table S4, PDF file, 0.02 MB. Copyright © 2019 Narrowe et al. 2019 Narrowe et al. This content is distributed under the terms of the Creative Commons Attribution 4.0 International license.

A soil slurry was made just prior to inoculation (*T*_0_) of microcosms and consisted of 1.125 g of anoxic soil in 25 ml of anaerobic, sterile water sealed in a serum vial with a butyl rubber stopper and an aluminum crimp under an atmosphere of 99.9% N_2_ gas. All microcosms containing soil were inoculated from this single soil slurry vial. Microcosms were inoculated at *T*_0_, and designated tubes were amended with 0.1 ml of a trimethylamine stock solution (4 mM) for a final concentration of 40 μM in the 10 ml of slurry. This first substrate addition (0.4 μmol) at a low concentration (0.004 μM) acted as a primer for methylotrophic methanogenesis, during a 2-day priming period (*T*_1_). Subsequently, 10 μmol of trimethylamine was added three times throughout the experiment (approximately every 7 days [*T*_2_ to *T*_4_] [Fig. 1]). Following each substrate addition, samples were taken for methane measurement and 16S rRNA analysis.

### Microcosm methane quantification.

Microcosm methane production was quantified at every microcosm time point (described above) using a Shimadzu (GC-2014) gas chromatograph (GC) equipped with a thermal conductivity detector using helium as a carrier gas at 100°C. All GC measurements are included in Table S1.

### Soil and porewater metabolite characterization.

Microcosm slurry and field soil porewater concentrations of formate, methanol, and mono-, di-, and trimethylamine were determined at the Pacific Northwest National Laboratory using proton nuclear magnetic resonance (^1^H NMR) as described in the work of Angle et al. (20), where concentrations of methanol in field soil porewater were previously reported. Soil acetate concentrations were determined using ion chromatography as reported by Angle et al. (20). Concentrations are reported as mean ± standard deviation.

### 16S rRNA amplicon sequencing and analysis.

Total genomic DNA was extracted from the microcosm slurries using the MoBio PowerSoil DNA isolation kit. Sequencing of the V4 region of the 16S rRNA gene was performed at Argonne National Laboratory’s Next Generation Sequencing Facility on the Illumina MiSeq using 251-bp paired-end reads and the Earth Microbiome Project primers 515F (5′-GTGCCAGCMGCCGCGGTAA-3′) and 806R (5′-GGACTACHVGGGTWTCTAAT-3′) (60). Reads were demultiplexed and analyzed within QIIME2 (80) (2017.10) using DADA2 (61) to produce an amplicon sequence variant (ASV) by sample table, filtered to retain only those sequences observed at least twice in any single sample (Data Set S1). The ASV table was imported into the Phyloseq package (62) for R and was used to calculate intersample Bray-Curtis dissimilarity followed by nonmetric dimensional scaling (NMDS) ordination and plotting using ggplot2 (63). Multiresponse permutation procedure (MRPP) tests for clustering of samples by microbial community composition were conducted using Vegan (64). The ASV table was summarized to taxon level 7 using QIIME2 and was used as input to LEfSe (65) to identify taxa which were discriminant in the inoculum and the TMA-amended samples. LEfSe calculated LDA greater than 2 is considered significant (65).

### *Methanomassiliicoccaceae* ASV meta-analysis.

ASVs assigned to the *Methanomassiliicoccaceae* were searched against the GenBank NT database (29 March 2019) as described previously (20). Briefly, each ASV was searched using BLASTN, with an E value of 1e−10 and -num_alignments 100000. Hits greater than 99% nucleotide identity over at least 200 bp were retained for further analysis. For these sequences, corresponding GenBank sequence deposition records were parsed to determine the environmental source of the sample in which the hit was identified.

### Metagenomic DNA sequencing, assembly, and analysis.

Total genomic DNA was extracted from the surface (0- to 5-cm) subsection of the soil core using the MoBio PowerSoil DNA isolation kit. Genomic DNA (with normal input concentrations ranging from 27 to 39 ng/μl) was prepared for shotgun metagenomic sequencing using the Kapa-Illumina library creation kit (Kapa Biosystems) and was sequenced at the Department of Energy Joint Genome Institute on the Illumina HiSeq2500. Fastq files were trimmed using Sickle (v 1.33) (66), and trimmed reads were assembled using IDBA-UD (67) using k-mers (40, 60, 80, and 100) as previously described (20). Genes were predicted using Prodigal (68) as part of an in-house annotation pipeline (https://github.com/TheWrightonLab/metagenome_analyses). In addition to the annotation results, we searched for *mcrA*, *mtaABC*, *mttBC*, *mtbBC*, and *mtmBC* genes using HMMER (69) against PFAM, TIGRFAM, and EGGNOG models (mcrA_C, PF02249; mcrA_N, PF02745; *mtaA*, TIGR01463; *mtaB*, PF12176 and ENOG410Y72C; *mtaC*, *mttC*, *mtbC*, and *mtmC*, TIGR02370; *mtbB*, PF09505; *mtmB*, PF05369; *mttB*, COG5598 and TIGR02369). Methanol dehydrogenase genes *xoxF* and *mxaFGJI* were searched by BLASTP against reference gene sequences and by hmmsearch against PFAM02315.

### Metatranscriptomic sequencing and analysis.

Total RNA was extracted from the surface (0- to 5-cm) and deep (24- to 35-cm) subsections of the soil core using the MoBio PowerSoil total RNA isolation kit. RNA was prepared and sequenced at the DOI Joint Genome Institute as described previously (20, 35). Briefly, sequencing libraries were prepared using the Illumina TruSeq Stranded Total RNA LT sample prep kit following rRNA depletion using the Illumina Ribo-Zero rRNA removal kit. All the plant surface sample RNA concentrations were normalized to 24 ng/μl as input concentration, and plant deep sample RNA concentrations were normalized to 15 ng/μl. Libraries were sequenced on the Illumina HiSeq 2500 using paired-end 150-bp reads (2 × 150). Fastq reads from triplicate (surface and deep samples) metatranscriptomes were trimmed using Sickle (v1.33) and mapped to predicted gene sequences, and the number of mapped transcripts per million (TPM) was calculated using Kallisto (v 0.45.0) (70). In order to ensure that deep soil transcript recruitment was not impacted by strain-level variation across surface and deep metagenomes, we expanded our target database to include genes predicted from metagenomic assemblies (scaffolds of >1 kb) representing multiple soil cover types, depths, and sampling years as described above. Genes with an average TPM of >2 across either the surface or the deep metatranscriptomes were retained for further analyses.

### *mcrA* gene phylogeny.

The *mcrA* sequence phylogeny was generated using a wetland *mcrA* gene database consisting of *Methanomassiliicoccaceae* genes identified from 6 surface soil metagenomes (20, 35) and 5 deep soil metagenomes collected from the same wetland transect 1 year prior to the sampling in this study (71) (Table S3). The phylogeny includes reference *mcrA* sequences (nucleotide) from genomes and metagenome-assembled genomes (MAGs) which comprised at least 34% of the full gene length. All GenBank assemblies within the *Methanomassiliicoccales* (24 July 2019) were searched for the *mcrA* gene using hmmsearch (PF02745). Genes sequences (nucleotide) with a minimum length of 750 nucleotides were retained for phylogenetic analyses. OWC sequences and genomic/MAG sequences were aligned using MUSCLE (v3.8.31) (72). The maximum-likelihood phylogenetic tree was generated using IQ-TREE (v1.5.5; -bb 1000 -alrt 1000) (73) with the model GTR+F+R6 chosen by Bayesian information criterion (BIC) using ModelFinder (74) within IQ-TREE with branch support calculated using UFBoot (75) and the SH-aLRT test (76). Transcripts from the triplicate surface and deep metatranscriptomes were mapped to the database as described previously (20, 35). The tree was visualized using iTOL (77) showing mean FPKM (fragments per kilobase of transcript per million reads mapped) values for each gene for each soil depth (surface *n* = 3 and deep *n* = 3).

### *mtaB* gene phylogeny.

All GenBank assemblies within the *Methanomassiliicoccales* (24 July 2019) were searched for the *mtaB* gene using hmmsearch against PFAM12176. Gene sequences coding for a minimum length of 225 amino acids (∼50% of full length) were retained for phylogenetic analyses. Transcribed OWC *mtaB* genes were also used in a search of the UniRef90 database. All BLASTP hits meeting an E value threshold of 1e−10 were incorporated in the phylogeny. Gene sequences were aligned using MAFFT (–auto) (78) and trimmed using TrimAl (-gappyout) (79). The maximum-likelihood phylogenetic tree was generated using IQ-TREE as described above, with the LG+F+R5 model selected by ModelFinder. The tree was visualized using iTOL (77).

### Data availability.

The metagenomic (surface soils) and metatranscriptomic sequencing data and the 16S rRNA amplicon sequencing reads for the microcosm experiment can be found in the NCBI Sequencing Read Archive under BioProject PRJNA338276 (20, 35). Metagenomic sequencing data for deep soil samples are found in the NCBI Sequencing Read Archive under identifiers SRX5010711, SRX5010712, SRX2839627, SRX3527544, and SRX3527565 (71). All SRA accession numbers can be found in Table S3.
