# Supplementary material for: Uncovering the Diversity and Activity of Methylotrophic Methanogens in Freshwater Wetland Soils
Source: mSystems. 2019 Dec 3;4(6):e00320-19. doi: 10.1128/mSystems.00320-19 (PMC6890927; doi:10.1128/mSystems.00320-19)
Supplement: TABLE S1 [file mSystems.00320-19-st001.pdf]

SOIL MICROCOSMS methane produced (µmol)				
	µmol Methane total			
Timepoint	T0	T2	T3	T4
Day	0	9	16	24
Soil TMA 1	BD	2.56	33.51	14.28
Soil TMA 2	1.55	3.16	17.42	100.30
Soil TMA 3	BD	1.32	5.57	76.28
Soil NO Substrate 1	BD	1.52	1.68	BD
Soil NO Substrate 2	BD	1.53	1.63	1.69
Soil NO Substarte 3	BD	1.36	1.41	1.39
