# Supplementary material for: Uncovering the Diversity and Activity of Methylotrophic Methanogens in Freshwater Wetland Soils
Source: mSystems. 2019 Dec 3;4(6):e00320-19. doi: 10.1128/mSystems.00320-19 (PMC6890927; doi:10.1128/mSystems.00320-19)
Supplement: TABLE S2 [file mSystems.00320-19-st002.pdf]

SOIL MICROCOSMS H-NMR detected metabolites (µM)

A

	Inoculum - T0 - after first addition of TMA			Final - T4				
	Soil_inoc_1	Soil_inoc_2	Soil_inoc_3	Soil_TF_NoSub2	Soil_TF_NoSub3	Soil_TF_TMA3	Soil_TF_TMA3	Soil_TF_TMA3
Acetate	8.1	11.5	7.3	13.3	42.4	297.5	238.0	187.9
Acetone	3.6	4.0	3.4	4.2	4.0	4.0	4.1	3.9
Butyrate	0.0	0.0	0.0	0.0	0.0	0.0	3.1	1.7
Ethanol	0.0	26.8	0.0	4.8	11.1	0.0	15.1	21.8
Formate	6.1	6.5	3.8	0.0	0.0	0.0	0.0	0.0
Isobutyrate	0.0	0.0	0.0	0.0	0.0	5.4	6.5	2.8
Isopropanol	0.0	1.6	0.8	0.7	1.2	0.7	1.4	1.8
Isovalerate	0.0	0.0	0.0	0.0	0.0	3.1	4.2	1.2
Methanol	0.0	4.3	1.0	0.0	1.4	0.0	1.4	2.0
Methylamine	0.0	0.0	0.0	0.0	0.0	0.0	0.0	49.5
Propionate	0.0	0.0	2.0	0.0	0.0	0.0	9.7	8.7
Trimethylamine	48.4	43.1	39.7	0.0	0.0	0.0	0.0	0.0

B

FIELD SOILS

	Soil Depth (cm)	*in situ* Temperature (˚C)	Dissolved Oxygen (µm)	Porewater (SED_IC) Acetate(mM)	H-NMR Methanol (uM)
August Plant Surface 1	5	25.4	164.90	0.61	65.601
August Plant Surface 2	5	27.4	104.40	0.51	39.6
August Plant Surface 3	5	25.0	62.00	0.47	25.086
August Plant Deep 1	25	24.1	47.90	0.24	1457
August Plant Deep 2	25	25.0	1.75	0.34	203.574
August Plant Deep 3	25	23.3	0.22	0.30	1305

Panel A shows the H-NMR results for the microcosm soils. Note that the T0 measurement was taken after the addition of TMA to verify the concentration of TMA added. Panel B shows the H-NMR and IC data for field soil for methanol and acetate as previously reported in Angle et al. 2017.
