# Supplementary figures and images for: Uncovering the Diversity and Activity of Methylotrophic Methanogens in Freshwater Wetland Soils
Source: mSystems. 2019 Dec 3;4(6):e00320-19. doi: 10.1128/mSystems.00320-19 (PMC6890927; doi:10.1128/mSystems.00320-19)

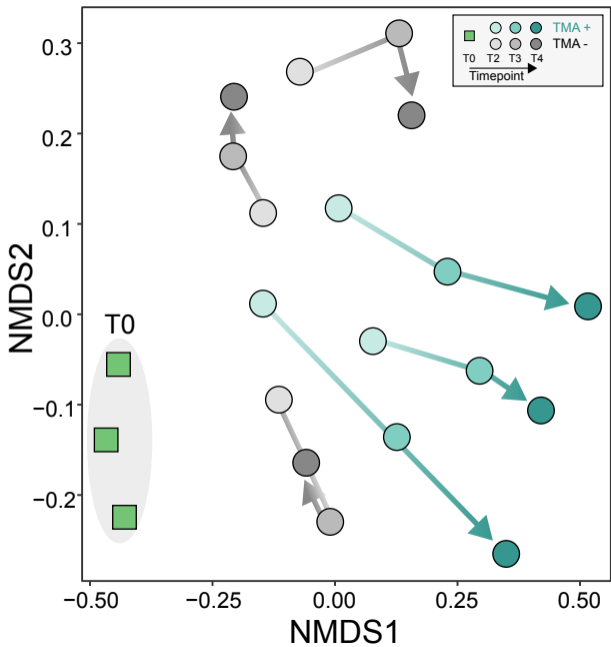

Supplement: FIG S1 [file mSystems.00320-19-sf001.pdf]
