# Supplementary material for: Uncovering the Diversity and Activity of Methylotrophic Methanogens in Freshwater Wetland Soils
Source: mSystems. 2019 Dec 3;4(6):e00320-19. doi: 10.1128/mSystems.00320-19 (PMC6890927; doi:10.1128/mSystems.00320-19)
Supplement: TABLE S3 [file mSystems.00320-19-st003.pdf]

Metagenomes used in gene databases

Name	SRA ID	Number of read pairs	Collection season	Ecosite type	Soil depth	Reference
August Plant Surface	SRX2995881	138290759	2014 - 15	Plant	0 - 5 cm	Angle et al. 2017
August Mud Surface	SRX2995880	115990605	2014 - 15	Mud flat	0 - 5 cm	Angle et al. 2017
August Open Surface	SRX2995879	115999605	2014 - 15	Water covered	0 - 5 cm	Angle et al. 2017
Nov Plant Surface	SRX2995884	98581091	2014 - 15	Plant	0 - 5 cm	Angle et al. 2017
Nov Mud Surface	SRX2995883	208578991	2014 - 15	Mud flat	0 - 5 cm	Angle et al. 2017
Nov Open Surface	SRX2995882	96172864	2014 - 15	Water covered	0 - 5 cm	Angle et al. 2017
M3C3D3_v1	SRX5010711	185599683	2013	Mud flat	13-23 cm	this study
M3C3D3_v2	SRX5010712	199616862	2013	Mud flat	13-23 cm	this study
M3C3D4	SRX2839627	208687232	2013	Mud flat	24- 35 cm	this study
O3C3D3	SRX3527565	179954643	2013	Water covered	13-23 cm	Narrowe, Spang et al. 2018
O3C3D4	SRX3527544	197763054	2013	Water covered	24- 35 cm	Narrowe, Spang et al. 2018

Metatranscriptomes used for transcript mappings

Name	SRA ID	Number of read pairs	Collection season	Ecosite type	Soil depth	Reference
August Plant Surface 1	SRX3032361	29671086	2014 - 15	Plant	0 - 5 cm	Angle et al. 2017
August Plant Surface 2	SRX3032360	32957111	2014 - 15	Plant	0 - 5 cm	Angle et al. 2017
August Plant Surface 3	SRX3032357	29358844	2014 - 15	Plant	0 - 5 cm	Angle et al. 2017
August Plant Deep 1	SRX3032356	78839804	2014 - 15	Plant	24- 35 cm	this study
August Plant Deep 2	SRX3032368	69168871	2014 - 15	Plant	24- 35 cm	this study
August Plant Deep 3	SRX3032367	84025658	2014 - 15	Plant	24- 35 cm	this study

Microcosm 16S rRNA amplicon sequencing

SampleID	SRA Accession	Number of read pairs	Sample type	Timepoint	Replicate	Reference
Soil_inoc_1	SRX5733045	13579	Soil inoculum	0	1	this study
Soil_inoc_2	SRX5733044	20215	Soil inoculum	0	2	this study
Soil_inoc_3	SRX5733043	17587	Soil inoculum	0	3	this study
Soil_T2_NoSub_1	SRX5733042	13648	Control	2	1	this study
Soil_T2_NoSub_2	SRX5733041	18285	Control	2	2	this study
Soil_T2_NoSub_3	SRX5733040	8785	Control	2	3	this study
Soil_T2_TMA_1	SRX5733039	11792	TMA-amended	2	1	this study
Soil_T2_TMA_2	SRX5733038	13993	TMA-amended	2	2	this study
Soil_T2_TMA_3	SRX5733037	12332	TMA-amended	2	3	this study
Soil_T3_NoSub_1	SRX5733036	3303	Control	3	1	this study
Soil_T3_NoSub_2	SRX5733030	12729	Control	3	2	this study
Soil_T3_NoSub_3	SRX5733029	13589	Control	3	3	this study
Soil_T3_TMA_1	SRX5733028	20700	TMA-amended	3	1	this study
Soil_T3_TMA_2	SRX5733027	14007	TMA-amended	3	2	this study
Soil_T3_TMA_3	SRX5733034	16782	TMA-amended	3	3	this study
Soil_TF_NoSub_1	SRX5733033	24773	Control	4	1	this study
Soil_TF_NoSub_2	SRX5733032	19016	Control	4	2	this study
Soil_TF_NoSub_3	SRX5733031	16537	Control	4	3	this study
Soil_TF_TMA_1	SRX5733026	41354	TMA-amended	4	1	this study
Soil_TF_TMA_2	SRX5733025	11048	TMA-amended	4	2	this study
Soil_TF_TMA_3	SRX5733035	17987	TMA-amended	4	3	this study
