# Supplementary material for: Uncovering the Diversity and Activity of Methylotrophic Methanogens in Freshwater Wetland Soils
Source: mSystems. 2019 Dec 3;4(6):e00320-19. doi: 10.1128/mSystems.00320-19 (PMC6890927; doi:10.1128/mSystems.00320-19)
Supplement: FIG S2 [file mSystems.00320-19-sf002.pdf]

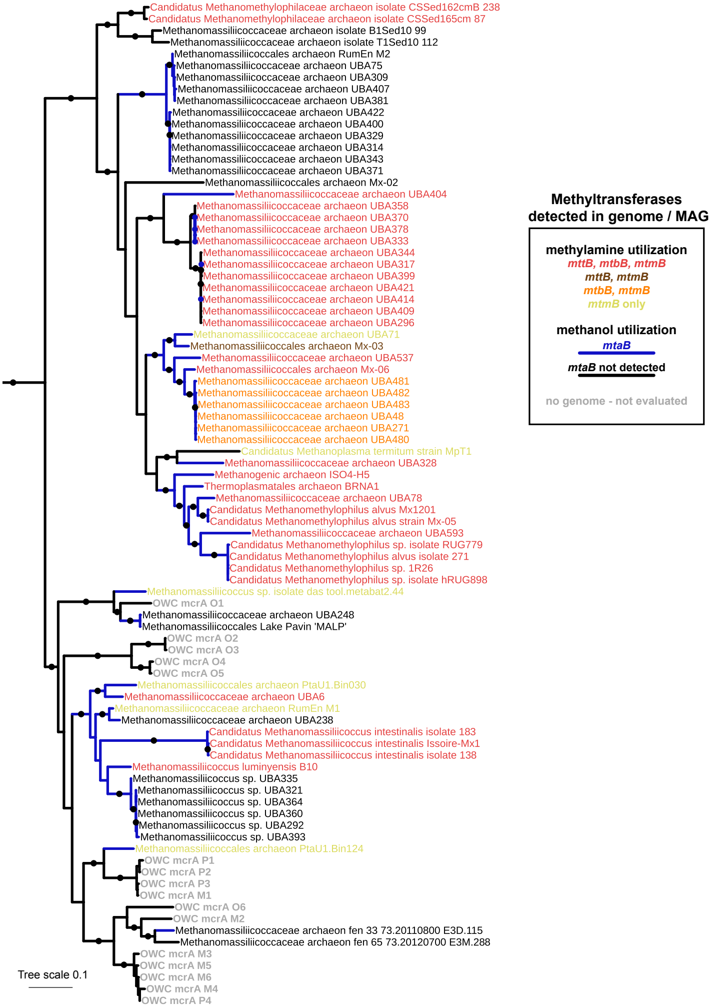

Candidatus Methanomethylophilaceae archaeon isolate CSSed162cmB 238
Candidatus Methanomethylophilaceae archaeon isolate CSSed165cm 87
Methanomassiliicoccaceae archaeon isolate B1Sed10 99
Methanomassiliicoccaceae archaeon isolate T1Sed10 112
Methanomassiliicoccales archaeon RumEn M2
Methanomassiliicoccaceae archaeon UBA75
Methanomassiliicoccaceae archaeon UBA309
Methanomassiliicoccaceae archaeon UBA407
Methanomassiliicoccaceae archaeon UBA381
Methanomassiliicoccaceae archaeon UBA422
Methanomassiliicoccaceae archaeon UBA400
Methanomassiliicoccaceae archaeon UBA329
Methanomassiliicoccaceae archaeon UBA314
Methanomassiliicoccaceae archaeon UBA343
Methanomassiliicoccaceae archaeon UBA371
Methanomassiliicoccales archaeon Mx-02
Methanomassiliicoccaceae archaeon UBA404
Methanomassiliicoccaceae archaeon UBA358
Methanomassiliicoccaceae archaeon UBA370
Methanomassiliicoccaceae archaeon UBA378
Methanomassiliicoccaceae archaeon UBA333
Methanomassiliicoccaceae archaeon UBA344
Methanomassiliicoccaceae archaeon UBA317
Methanomassiliicoccaceae archaeon UBA399
Methanomassiliicoccaceae archaeon UBA421
Methanomassiliicoccaceae archaeon UBA414
Methanomassiliicoccaceae archaeon UBA409
Methanomassiliicoccaceae archaeon UBA296
Methanomassiliicoccaceae archaeon UBA71
Methanomassiliicoccales archaeon Mx-03
Methanomassiliicoccaceae archaeon UBA537
Methanomassiliicoccales archaeon Mx-06
Methanomassiliicoccaceae archaeon UBA481
Methanomassiliicoccaceae archaeon UBA482
Methanomassiliicoccaceae archaeon UBA483
Methanomassiliicoccaceae archaeon UBA48
Methanomassiliicoccaceae archaeon UBA271
Methanomassiliicoccaceae archaeon UBA480
Candidatus Methanoplasma termitum strain MpT1
Methanomassiliicoccaceae archaeon UBA328
Methanogenic archaeon ISO4-H5
Thermoplasmatales archaeon BRNA1
Methanomassiliicoccaceae archaeon UBA78
Candidatus Methanomethylophilus alvus Mx1201
Candidatus Methanomethylophilus alvus strain Mx-05
Methanomassiliicoccaceae archaeon UBA593
Candidatus Methanomethylophilus sp. isolate RUG779
Candidatus Methanomethylophilus alvus isolate 271
Candidatus Methanomethylophilus sp. 1R26
Candidatus Methanomethylophilus sp. isolate hRUG898
Methanomassiliicoccus sp. isolate das tool.metabat2.44
OWC mcrA O1
Methanomassiliicoccaceae archaeon UBA248
Methanomassiliicoccales Lake Pavin 'MALP'
OWC mcrA O2
OWC mcrA O3
OWC mcrA O4
OWC mcrA O5
Methanomassiliicoccales archaeon PtaU1.Bin030
Methanomassiliicoccaceae archaeon UBA6
Methanomassiliicoccaceae archaeon RumEn M1
Methanomassiliicoccaceae archaeon UBA238
Candidatus Methanomassiliicoccus intestinalis isolate 183
Candidatus Methanomassiliicoccus intestinalis Issoire-Mx1
Candidatus Methanomassiliicoccus intestinalis isolate 138
Methanomassiliicoccus luminyensis B10
Methanomassiliicoccus sp. UBA335
Methanomassiliicoccus sp. UBA321
Methanomassiliicoccus sp. UBA364
Methanomassiliicoccus sp. UBA360
Methanomassiliicoccus sp. UBA292
Methanomassiliicoccus sp. UBA393
Methanomassiliicoccales archaeon PtaU1.Bin124
OWC mcrA P1
OWC mcrA P2
OWC mcrA P3
OWC mcrA M1
OWC mcrA O6
OWC mcrA M2
Methanomassiliicoccaceae archaeon fen 33 73.20110800 E3D.115
Methanomassiliicoccaceae archaeon fen 65 73.20120700 E3M.288
OWC mcrA M3
OWC mcrA M5
OWC mcrA M6
OWC mcrA M4
OWC mcrA P4
Tree scale 0.1
Methyltransferases detected in genome / MAG
methylamine utilization
mttB, mtbB, mtmB
mttB, mtmB
mtbB, mtmB
mtmB only
methanol utilization
mtaB
mtaB not detected
no genome - not evaluated
