# Supplementary material for: Uncovering the Diversity and Activity of Methylotrophic Methanogens in Freshwater Wetland Soils
Source: mSystems. 2019 Dec 3;4(6):e00320-19. doi: 10.1128/mSystems.00320-19 (PMC6890927; doi:10.1128/mSystems.00320-19)
Supplement: TABLE S4 [file mSystems.00320-19-st004.pdf]

Vitamin Mix

Components	Amount mg L^{-1} ddi H_2O
d-Biotin	2
Folic Acid	2
Pyridoxine HCl	10
Riboflavin	5
Thiamine	5
Nicotinic Acid	5
Pantothenic Acid	5
Vitamin B12	0.1
p-Amino Benzoic Acid	5
D,L-6,8-Thiotic Acid	5

Mineral Mix

Components	Amount g L^{-1} ddi H_2O
NTA Disodium Salt	1.5
$MgSO_4 \cdot 7H_2O$	3
$MnSO_4 \cdot H_2O$	0.5
NaCl	1.0
$FeSO_4 \cdot 7H_2O$	0.1
$CaCl_2 \cdot 2H_2O$	0.1
$CoCl_2 \cdot 6H_2O$	0.1
ZnCl	0.13
$CuSO_4 \cdot 5H_2O$	0.01
$AlK(SO_4)_2 \cdot 12H_2O$	0.01
Boric Acid	0.01
$Na_2MoO_4 \cdot 2H_2O$	0.025
$NiCl_2 \cdot 6H_2O$	0.024
$Na_2WO_4 \cdot 2H_2O$	0.025
Na_2SeO_4	0.02
